# Evaluation of *Salud Para Su Corazón* (Health for Your Heart) — National Council of La Raza Promotora Outreach Program

**Published:** 2005-06-15

**Authors:** Héctor Balcázar, Matilde Alvarado, Mary Luna Hollen, Gonzalez-Cruz Yanira, Verónica Pedregón

**Affiliations:** University of Texas Health Science Center at Houston, School of Public Health, El Paso Regional Campus; National Heart, Lung, and Blood Institute, National Institutes of Health, Bethesda, Md; University of North Texas Health Science Center at Fort Worth, School of Public Health, Department of Social & Behavioral Sciences, Fort Worth, Tex; National Hispanic Council on Aging, Washington, DC; University of Texas Health Science Center at Houston, School of Public Health, El Paso Regional Campus, El Paso, Tex

## Abstract

**Introduction:**

In 2001, the National Heart, Lung, and Blood Institute partnered with the National Council of La Raza to conduct a pilot test of its community-based outreach program *Salud Para Su Corazón* (Health for Your Heart), which aims to reduce the burden of morbidity and mortality associated with cardiovascular disease among Latinos.

**Methods:**

The effectiveness of *promotores de salud* (community health workers) in improving heart-healthy behaviors among Latino families participating in the pilot program at seven sites was evaluated. Data on the characteristics of the *promotores* in the *Salud Para Su Corazón* program were compiled. *Promotores* collected data on family risk factors, health habits, referrals and screenings, information sharing, and program satisfaction from 223 participating Latino families (320 individual family members) through questionnaires. Paired *t* tests and chi-square tests were used to measure pretest–posttest differences among program participants.

**Results:**

Results demonstrated the effectiveness of the *promotora* model in improving heart-healthy behaviors, promoting community referrals and screenings, enhancing information sharing beyond families, and satisfying participants' expectations of the program. The main outcome of interest was the change in heart-healthy behaviors among families.

**Conclusion:**

The community outreach model worked well in the seven pilot programs because of the successes of the *promotores* and the support of the community-based organizations. Successes stemmed in part from the train-the-trainer approach. *Promotoria*, as implemented in this program, has the potential to be integrated with a medical model of patient care for primary, secondary, and tertiary prevention.

## Introduction

Recent epidemiological studies have demonstrated that Latinos may not be as protected from mortality events associated with cardiovascular disease (CVD) as postulated in earlier studies (1). Compared with non-Hispanic whites, Mexican Americans, who represent the largest percentage of the U.S. Hispanic population, experience equal or greater rates of CVD mortality (2,3). This disparity is paralleled by the increased prevalence of associated risk factors (e.g., obesity, diabetes, lack of physical activity) for CVD in this and other Hispanic populations (4). The response to combat CVD and its risk factors among Hispanics necessitates the development of culturally competent programs that include community outreach strategies that target underserved and underinsured Hispanic populations. The *Salud Para Su Corazón* (*SPSC*) (Health for Your Heart) program is one example of a community-based outreach strategy that aims to reduce CVD risk factors and ultimately reduce the burden of mortality associated with CVD among Latinos.

*SPSC* was developed in 1994 by the National Heart, Lung, and Blood Institute (NHLBI) of the National Institutes of Health (NIH) as a community-based health promotion model for heart health among Latinos (5-9). The integration of the *promotora de salud* (community health worker) model distinguishes *SPSC* from other heart health programs. The use of community health workers has proven to be a cost-effective intervention strategy for expanding health access and health care services to underserved and underinsured minority communities, including Latino communities (10).

In 2001, the NHLBI partnered with the National Council of La Raza (NCLR), the nation's largest Hispanic grassroots organization, to establish a national pilot dissemination of *SPSC* programs. The partnership sought to test the *promotora* model for promoting heart health and reducing CVD risk factors within seven Latino communities across the country. *Promotores de salud* and community-based organizations (CBOs) teamed up to deliver the culturally competent *SPSC* health promotion intervention.

The *SPSC*–NCLR initiative to test the *promotora* outreach model had three components: 1) a planning and development phase, 2) an implementation phase, and 3) an evaluation phase. The description of the planning and development phase has been published elsewhere (11). The objectives of the implementation and evaluation phases for the *SPSC*–NCLR initiative were to 1) monitor changes in heart health knowledge, attitudes, and skills of *promotores*; 2) track the delivery of program curriculum and educational sessions by *promotores* to Latino families; 3) deliver a series of community-educational activities; 4) assess perceptions of CBOs associated with the *promotora* model to build community capacity; and 5) evaluate changes in heart-healthy behaviors reported by Latino family participants. This article describes the *SPSC*–NCLR pilot dissemination approach, focusing on the implementation and evaluation phases of the initiative, and describes the effectiveness of *promotores* to improve heart-healthy behaviors among participant Latino families.

## Methods

### The S*PSC–*NCLR pilot dissemination approach

The theoretical framework guiding the *SPSC*–NCLR initiative integrated two major elements: community outreach processes and participatory research methods. This integrated approach guided the development, implementation, and evaluation of the train-the-trainer *promotora* model.

### Development of the *promotora* model

The development phase of the *SPSC-*NCLR initiative consisted of a needs assessment survey of CBOs, which was used for selecting the seven participating sites. These participating sites were CBOs that had *promotores* working for them. The Table provides the names and locations of the CBOs. A detailed description of the development of this model is described elsewhere (11).

### Implementation of the *promotora* model

The implementation phase at each site consisted of 1) *promotora* training activities, 2) activities to recruit participant Latino families, and 3) a 6-month intervention delivered by *promotores*.

#### *Promotora* training.

A series of well-defined and structured *promotora* training activities was conducted and supervised by the *SPSC*–NCLR team for all seven sites. The activities involved the development of participatory process and capacity building to support the adequate delivery of the interventions (11).

#### Recruitment of Latino families.

Recruitment was primarily the responsibility of the *promotores*. They recruited families using advertisements developed by their own CBOs, and they enrolled participants from community centers, health centers, schools, and other community and neighborhood sites. *Promotores* approached individual family members and invited them to participate in the program. All family members were invited to participate in the program; however, the mother was the most likely participant (91% of the time).

#### Intervention.

The intervention implemented at all seven sites included a series of educational sessions from the NHLBI heart-health curriculum called *Your Heart, Your Life*. *Promotores* delivered seven of eight curriculum lessons in 2-hour sessions within a 2- to 3-month period for the first part of a 6-month intervention. The eighth lesson on diabetes was optional. Sessions occurred either several times a week, once a week, or every other week. *Promotores* used a variety of educational materials including workbooks, *fotonovela* stories, easy-to-read booklets, and videos. The intervention also included home-visit or telephone follow-up contacts to reinforce the educational activities learned in the program. More detailed information about this family heart-health education program using *Your Heart, Your Life* has been reported in a recent article (11). Additional activities under the contracts for the CBOs and their *promotores* included referrals to health care providers for health screenings of blood pressure, blood cholesterol, blood glucose, and measures of weight and waist circumference.

The *SPSC*–NCLR program reached 223 Latino families served by 33 *promotores* at seven locations across the United States. The majority of the families served by the program resided in California, Illinois, New Mexico, and Texas and were of Mexican origin. Latino families of Central and South American origin were also represented in the communities of Chicago, Ill; Escondido, Calif; and Providence, RI.

### Evaluation of the *promotora* model

The evaluation framework focused on the content of the program, how it was implemented, and its results. To evaluate the extent to which the *SPSC*-NCLR program effected change in awareness, knowledge, and behavior, data were collected using an evaluation tool called *¡Cuéntamelo!* (Tell me about it!) at three levels: 1) the CBOs, 2) the trained *promotores*, and 3) the participating families. Designed to guide and assess program implementation, *¡Cuéntamelo!* captures process and outcome data with several instruments. Data sources include questionnaires completed by *promotores*, families, and CBOs as well as interviews. A full description of the instruments comprising *¡Cuéntamelo!* (11) as well as evaluation data provided by participating CBOs (12) are published elsewhere.

This evaluation consisted of analyzing data collected from 33 *promotores* and 223 families (including a total of 320 individual family members) across all seven Latino communities. The evaluation focused on the characteristics of the *promotores* participating in the pilot program; the impact of the intervention on improving heart-healthy behaviors among family participants; the levels of referrals and screenings resulting from the program; the extent to which family participants shared their newly gained knowledge with others; and degree of participant satisfaction with the program.

### Data analysis

Questionnaires were designed to facilitate data entry into a computer database using Epi Info 2002 (Centers for Disease Control and Prevention, Atlanta, Ga). The SPSS statistical software program (SPSS Inc, Chicago, Ill) was used to do a variety of analyses, which included calculating frequencies of responses to each question, computing averages and scores, and comparing responses across families and *promotores*. Paired *t* tests and chi-square tests were used to test for pre–post differences in family habits. Average improvement scores based on pre–post tests and their 95% confidence intervals were also calculated. Data analysis is presented for all sites combined based on several factors: 1) 91% of participants were women; 2) the mean age of participants for each site ranged from 30 to 51 years; 3) data on country of origin were not available for subgroup analysis; and 4) sample size was limited for some sites.

The main outcome variable of interest was the change in heart-healthy behaviors among family contact persons. A pre–post research design without a control group was used because the outreach programs were pilot programs and because of financial and logistic limitations. Families participating in the *Your Heart, Your Life* educational sessions completed a survey on practices of heart-healthy behaviors both before and after the sessions. The average time between the completion of the curriculum and the posttest was 3 months. A 35-item self-report 4-point scale for assessing family habits (0 = never, 3 = always) was used to assess heart-healthy behaviors.

The family habits scale included many items that assessed the frequency with which families engaged in a variety of heart-healthy practices, such as exercise and eating a low-sodium diet. Two groups of physical activity questions were asked. For the question "What does the family do to be more active?," the possible responses included such activities as walking, dancing, riding a stationary bike, working in the garden, aerobic dancing, or playing soccer. The second group of physical activity questions focused on such activities as getting off a bus one or two stops early and walking and using stairs instead of elevators. Families could choose to answer "never," "sometimes," "usually," or "always." Responses were converted for each of the items representing a desirable behavior to a 0–3-point scale with "never" equaling 0 and "always" equaling 3, assuming there were equal distances between responses. Subscores were calculated by adding responses to individual items on the same topic. An overall score using responses to all items was also computed. For ease of interpretation, scores were converted to a 0–100-point scale in which a high score reflected a higher frequency of always practicing a desirable behavior. Content validity and reliability of these subscales have been reported elsewhere (11,13) and shown to have good reliability (Cronbach α > 0.60) (13).

## Results

### Promotores

A total of 33 *promotores* delivered the intervention. The average number of families per *promotor(a)* was seven, with a range of 2 to 13 families per *promotor(a)*. The Table presents the number of *promotores* working with participant families, the total number of families, and the mean number of families per *promotor(a)* at each site.

Most *promotores* were women aged 20 to 67; the average age was 41. About 65% of the *promotores* were born outside the United States. Of the 33 *promotores*, 5% reported having attended only primary school or less, 18% reported having some high school education, 28% reported having a high school diploma or GED, 30% reported having some college or technical school education, and 18% reported being college graduates. Overall, most *promotores* reported being bilingual. As would be expected, the preferred language varied depending on whether the *promotores* were born in the United States; a greater proportion preferred English or English and Spanish if they were born in the United States. Most *promotores* (78%) had worked as lay health educators before participating in the *SPSC*-NCLR program, with experience ranging from 6 months to 20 years.

### Improving heart health of families

The location for conducting family education sessions varied slightly depending on the topic; the most common locations were family homes (35%) and community centers (32%). Other locations (e.g., church, school) were also used for about 27% of the family education sessions. Fewer than 5% of the family education sessions were conducted in health centers. The surveyed families received an average of about seven educational sessions out of the possible eight. Almost all families (89%) were given information on at least seven topics, including diabetes.

*Promotores* asked the main respondent for the family, usually the mother, to provide the estimated number of individuals in the family at risk for CVD. Among the main respondents for the 223 families, 57% reported that no one in their family was at risk for CVD; 42% reported that they had at least one family member at risk.

**Figure 1:**
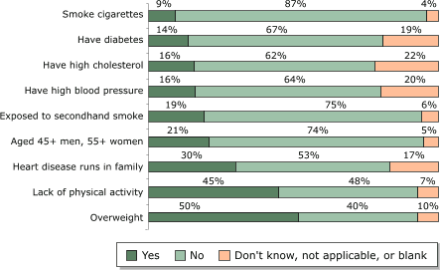
Heart disease risks reported by individual family members (n = 320) participating in the *Salud Para Su Corazón* outreach program. Data collected by *promotores* using questionnaires, 2001.

Figure 1 shows CVD risks reported by individual family members. When *promotores* asked individual family members to report on a list of risk factors, 80% of the 320 individuals surveyed had at least one risk for heart disease. The mean number of risks per family member surveyed, excluding those who reported having no risks, was three. The overall mean number of risks, including the individuals who reported having no risks, was two. The two most common risks factors for CVD among all individuals surveyed were being overweight (50%) and a lack of physical activity (45%). Only 9% of the respondents reported that they smoked, a low percentage compared with other factors.

**Table:** 

	**Percentage of families reporting on heart disease risks, by response**		

	**Yes**	**No**	**Don't know, not applicable, or blank**
Smoke cigarettes	9	87	4
Have diabetes	14	67	19
Have high cholesterol	16	62	22
Have high blood pressure	16	64	20
Exposed to secondhand smoke	19	75	6
Aged 45+ men, 55+ women	21	74	5
Heart disease runs in family	30	53	17
Lack of physical activity	45	48	7
Overweight	50	40	10

**Figure 2:**
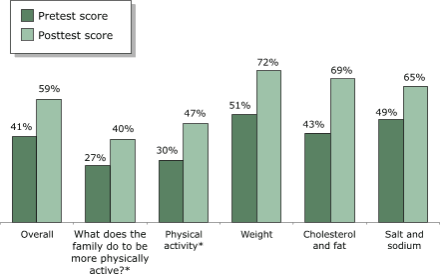
Average scores, by topic, for heart-healthy habits among families (n = 190) participating in both the pretest and posttest as part of the *Salud Para Su Corazón* outreach program. Score percentages calculated based on maximum score of 100. Asterisk (*) indicates that data refer only to mother. *P* < .001 for paired *t* tests on all topics. Data collected by *promotores* using questionnaires, 2001.

Changes in pretest and posttest scores for the families' practices of heart-healthy behaviors are presented in Figure 2. Families showed improvement in heart-healthy behaviors. Paired *t* tests of the increases in average overall scores and for each topic were statistically significant (*P* < .001). For the 190 families that completed both surveys, the average pretest overall score was 41% and the average posttest overall score was 59%, representing an average improvement in the total score of 18% (95% confidence interval [CI], 16%–21%).

**Table:** 

	**Pretest Score**	**Posttest Score**
Overall	41	59
What does the family do to be more physically active?*	27	40
Physical activity*	30	47
Weight	51	72
Cholesterol and fat	43	69
Salt and sodium	49	65

The greatest improvement was observed for the items on practices related to cholesterol and fat, with an average pretest score of 43% and an average posttest score of 69%, an improvement of 26% (95% CI, 23%–30%). On topics such as "What is the family doing to be more physically active?" an average improvement of 13% was found (95% CI, 9%–16%). An improvement of 17% (95% CI, 14%–21%) was found for the physical activity topic. An average improvement of 21% (95% CI, 18%–24%) was found in practices related to weight reduction and control. Improvements were also observed on items related to salt and sodium consumption.

**Figure 3:**
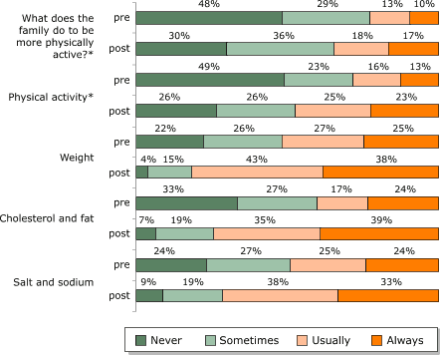
Distribution of responses on family heart-healthy habits among families (n = 190) participating in both the pretest and posttest as part of the *Salud Para Su Corazón* outreach program. Asterisk (*) indicates that data refer only to mother. Data collected by *promotores* using questionnaires, 2001. Percentages do not add to 100% because of rounding.

Figure 3 describes the distribution of responses across the four possible response categories. Chi-square tests of changes in the distribution were statistically significant (*P* < .001), even though this test does not correct for a potential underestimation of the effect due to the correlation between pretest and posttest scores for individual respondents.

**Table:** 

** **		**Percentage of families practicing heart-health behaviors, by frequency**			

		**Never**	**Sometimes**	**Usually**	**Always**
What does the family do to be more physically active?*	Pretest	48	29	13	10
	Posttest	30	36	18	17
Physical activity*	Pretest	49	23	16	13
	Posttest	26	26	25	23
Weight	Pretest	22	26	27	25
	Posttest	4	15	43	38
Cholesterol and fat	Pretest	33	27	17	24
	Posttest	7	19	35	39
Salt and sodium	Pretest	24	27	25	24
	Posttest	9	19	38	33

### Community referrals and screenings

Of the 223 families surveyed, *promotores* referred 74% to health care providers for blood pressure screening and 81% for blood cholesterol screening. They also weighed and measured the waist circumferences of family members for 77% of the families. Among the 101 families that received the educational session on diabetes, 70% were referred for a blood glucose check. According to the data collected by *promotores* through the group education questionnaires, *promotores* referred participants in 89% of the 56 classes taught on cholesterol for blood cholesterol screening and referred participants in 86% of the 37 classes taught on diabetes for blood glucose screening. *Promotores* also measured the weight and waist circumferences in 34% of the 32 classes taught on maintaining a healthy weight. No data were collected for blood pressure screenings.

Based on the *promotores* interviews conducted in 2003, five of the seven sites reported that more than 90% of the participants referred for screening in 2001 were actually screened, and one of the sites reported that 100% of the participants referred were screened. Another site reported that it did not have this information. To ensure that the participants would be screened after taking the class, some of the *promotores* opted for providing transportation to a clinic after the class, while others decided to bring a nurse to the session to do the screening. In other locations, *promotores* kept track of the percentage of participants who were screened by asking them for the screening results during follow-up visits. In still other locations, *promotores* arranged for free screening of class participants in an effort to increase the number of people getting screened for the different heart risk factors. The interaction between *promotora* and screening activities on reducing CVD risk factors was not explored because of limitations in data collection.

### Reaching beyond families

**Figure 4:**
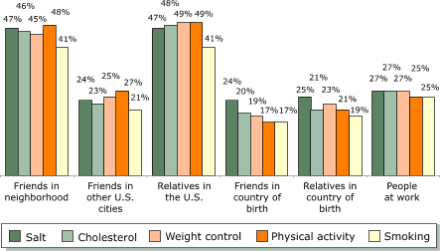
Audiences with whom families participating in *Salud Para Su Corazón* outreach program (n = 223) shared educational information received, by topic. Data collected by *promotores* using questionnaires, 2001.

According to the data collected from 223 families, *SPSC*–NCLR reached other people beyond the original participant families. Responses to questions on information sharing revealed that family respondents were likely to share information they learned about heart-healthy behaviors with friends in their neighborhood, friends in other cities, friends in their country of birth, relatives in their country of birth, or people at work. The results showed that family respondents were just as likely to share information about one topic as another, with the exception of smoking (Figure 4). Fewer than 33% of families shared information with friends in other cities, friends in their country of birth, relatives in their country of birth, or people at work. The lower proportion of families that shared information about smoking, compared with other topics, may reflect the fact that most respondents reported being nonsmokers.

**Table:** 

	**Percentage of families who shared information, by topic**				

**Audience with whom information was shared**	**Salt**	**Cholesterol**	**Weight control**	**Physical activity**	**Smoking**
Friends in neighborhood	47	46	45	48	41
Friends in other U.S. cities	24	23	25	27	21
Relatives in the U.S.	47	48	49	49	41
Friends in country of birth	24	20	19	17	17
Relatives in country of birth	25	21	23	21	19
People at work	27	27	27	25	25

### Participants' evaluations of *SPSC*–NCLR programs

**Figure 5:**
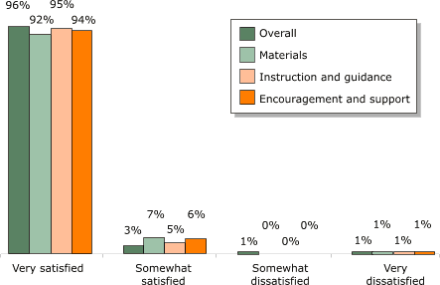
Percentages of families satisfied with guidance provided by the *Salud Para Su Corazón* outreach program (n = 207). Data collected by *promotores* using questionnaires, 2001.

Family participants expressed a very high level of satisfaction with the program. Overall, of the 207 families participating in the survey on satisfaction, 96% were very satisfied with the program. Accordingly, the level of satisfaction with specific aspects of the program was also very high (Figure 5). For example, the majority of the families surveyed (95%) rated the information they received through the program (instruction and guidance) as very important, including information on cholesterol and fat, blood pressure, weight control and serving information, physical activity, smoking, and salt and sodium.

**Table:** 

	**Percentages of families satisfied with program guidance, by satisfaction level**			

	**Very satisfied**	**Somewhat satisfied**	**Somewhat dissatisfied**	**Very dissatisfied**
Overall	96	3	1	1
Materials	92	7	0	1
Instruction and guidance	95	5	0	1
Encouragement and support	94	6	0	1

## Discussion

### Successes

The community outreach model worked well in the seven pilot programs because of the successes of the *promotores de salud* and the support of the CBOs (12). With their skills, commitment, and enthusiasm, the training program and health education curriculum proved effective, translating into success for the *SPSC*-NCLR initiative. Overall, the results of the evaluation show that the initiative resulted in significant accomplishments at all three levels: *promotores*, families, and CBOs (12).

The successes realized in promoting heart health stems in part from the train-the-trainer approach. It nurtured the competency of the *promotores* to ensure success of the intervention. The train-the-trainer approach empowered *promotores* in their work by broadening their range of instructional approaches and heart-health knowledge. Based on self-reports by *promotores* and families, after completing the *SPSC* training, *promotores* were able to obtain the knowledge and skills necessary to recruit community members to participate in the program, to pass on the knowledge they had gained, and to support community members in accomplishing lifestyle changes that promoted better heart health. *Promotores* also expanded awareness of the project in the community, solicited additional funding, developed public service announcements related to the teaching materials, or did all of these. Despite facing a number of challenges, including limited funding and the need to prove the value of their work and that of the *SPSC*-NCLR program to some health care professionals, they successfully established partnerships with a variety of local organizations and programs, including clinics and health care providers, churches, schools, radio stations, health professional associations, restaurants, and pharmaceutical companies.

*Promotoria*, as implemented in the *SPSC*-NCLR program, has the potential to be integrated with a medical model of patient care for primary, secondary, and tertiary prevention. Using the *¡Cuéntamelo!* evaluation tools, *promotores* successfully identified risks for heart disease at the levels of both families and individuals, referring individuals for screening and providing community members with the knowledge and skills that they needed to improve their heart health. The capacity of *promotores* to function as health educators and advocates has important implications for health promotion and disease prevention, given recent evidence that physicians spend less than 10 minutes per patient delivering educational messages (15).

The ability of *promotores* to effect behavioral change is evident among the families they served. Evidence suggests families participating in the *SPSC*-NCLR program made positive changes in heart-healthy behaviors. Families showed an improvement of 18% on self-reported heart-healthy behaviors. It seems that *promotores* succeeded at improving heart-health awareness and creating a cultural environment for learning heart-health information to promote changes in lifestyle behaviors among family members. Changes in heart-health behaviors have been consistently observed for similar *promotora* educational programs, adding credibility to the *promotora* approach (16,17).

According to the CBO survey (11,12), these local organizations supported the *SPSC*-NCLR program for two main reasons: the program supported the organization's mission of enhancing health education and prevention in Latino communities, and the program facilitated partnerships and provided access to additional human and financial resources. Furthermore, the adaptability of the *SPSC*-NCLR program facilitated its implementation at all seven sites. Even though all the sites applied the same *SPSC*-NCLR program training and materials (including the *¡Cuéntamelo!* tools), each site adapted some aspects of the program based upon their needs, the resources available, and the characteristics of the community. For example, some sites focused on conducting group education sessions; others focused on home visits for family education. Thus, *SPSC*-NCLR appears to be a flexible and adaptable program for use in a variety of settings and with different types of Latino communities (5,13,17,18).

### Recommendations

Four recommendations are identified to improve the implementation and evaluation of the *SPSC*-NCLR program. First, it might be useful to explore how to facilitate health screening and follow-up care for individuals whom *promotores* identify as being at risk for heart disease. The second recommendation follows from the difficulties reported by *promotores* in using videos as educational materials. These difficulties resulted from a lack of video equipment on site. It might be useful instead to use sociodramas based on the video scripts as supplemental educational tools or to replace the video entirely. The use of the videos could also be tested for its effectiveness in promoting awareness and changing behavior. Third, a distance-training and continuing-education module of *promotora* training can be considered, using a Web-based curriculum. It would be interesting to examine whether this form of training is effective; it might have implications for international use. Finally, a formal evaluation of the *SPSC*-NCLR program requires a quasiexperimental design or a clinical trial with pretest and posttest comparisons. It should involve evaluation of a series of sociodemographic and acculturation indicators, in addition to biological parameters, including changes in blood pressure, blood cholesterol, and body mass index. Finally, a formal evaluation should include more distal or secondary outcomes such as screening rates, sales of low-fat foods, and similar aspects in the community at large to indicate whether heart-healthy behaviors are becoming a community norm.

## Figures and Tables

**Table:** Sites and Numbers of Participants and *Promotores* for *Salud Para Su Corazón* Program

**Site of *Salud Para Su Corazón* Program**	**No. *Promotores***	**No. Participating Families**	**No. Families per *Promotora***
Centro San Bonifacio, Chicago, Ill	10	76	7.6
Centro San Vicente, El Paso, Tex	1	2	2.0
Neighborhood Health Care, Escondido, Calif	4	38	9.5
Hands Across Cultures, Española, NM	3	36	12.0
CHisPA, Providence, RI	4	10	2.5
Council for the Spanish Speaking, Stockton, Calif	5	32	6.4
Migrant Health Promotion, Las Colonias, Tex	6	29	4.8
**Total**	33	223	6.8
